# Supplementary material for: αv integrins on mesenchymal cells regulate skeletal and cardiac muscle fibrosis
Source: Nat Commun. 2017 Oct 24;8:1118. doi: 10.1038/s41467-017-01097-z (PMC5653645; doi:10.1038/s41467-017-01097-z)
Supplement: Supplementary file 1 — Supplementary Information [file 41467_2017_1097_MOESM1_ESM.pdf]

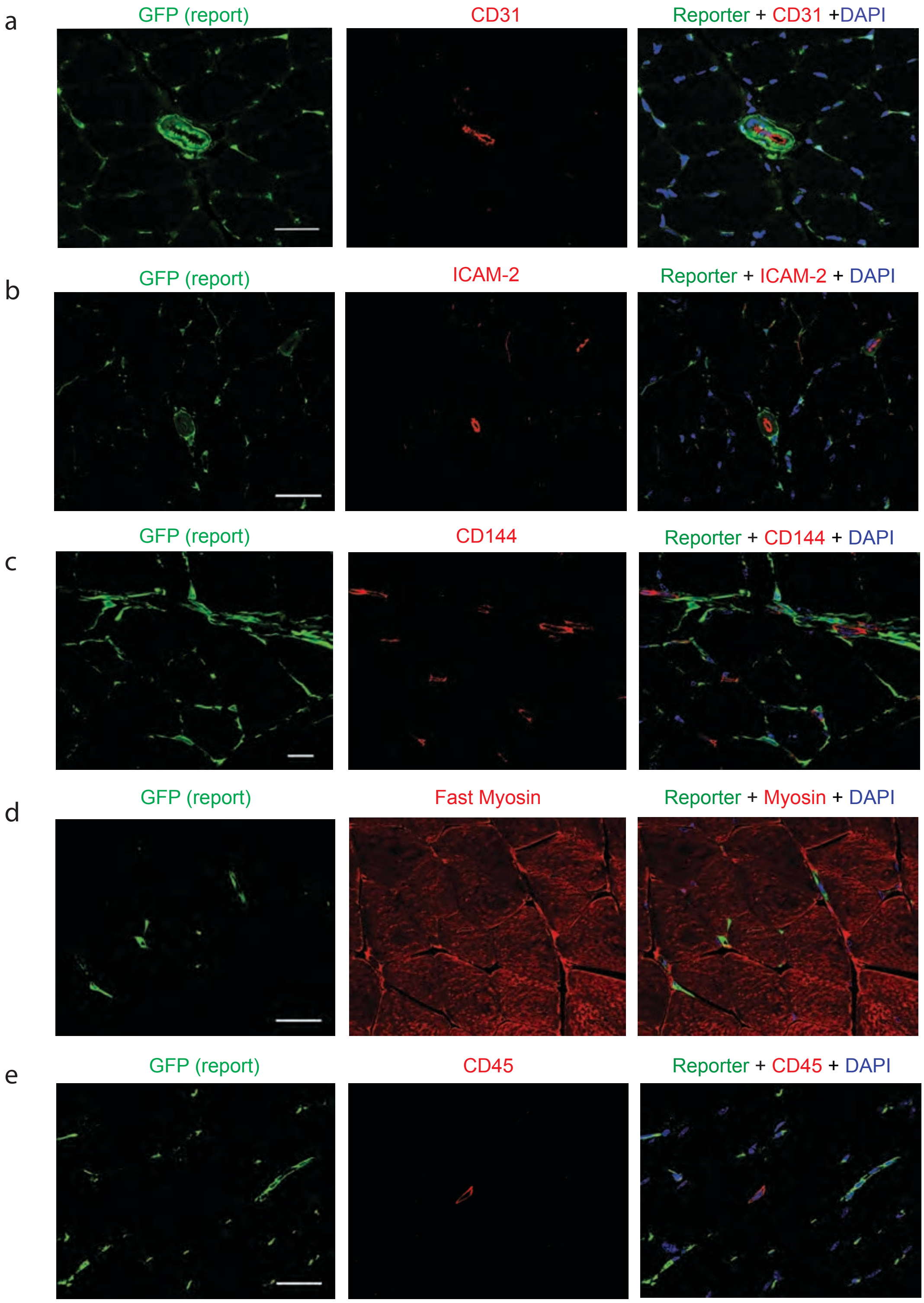

Supplementary Figure 1. Immunofluorescence micrographs of skeletal muscle sections from mTmG;PDGFR β-Cre reporter mice (n=4) stained with anti-CD31 (a) (scale bar 30μm), (b) anti-ICAM-2 (scale bar 30μm), (c) anti-CD144 (scale bar 15μm), (d) anti-myosin (scale bar 30μm), and (e) anti-CD45 (scale bar 30μm).

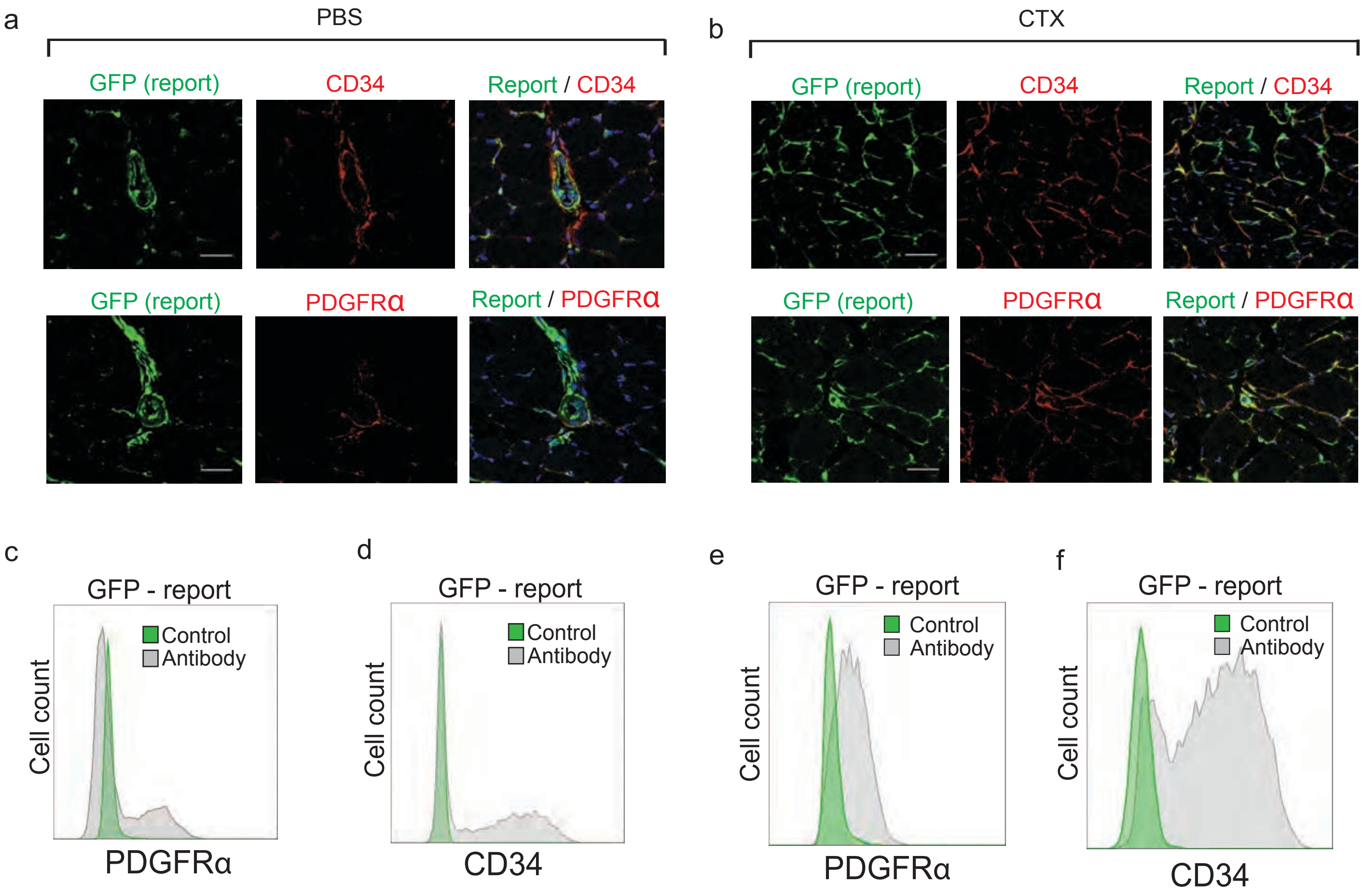

Supplementary Figure 2. Expression of PDGFRα and CD34 in PDGFRβ⁺ reporter cell populations from mTmG;PDGFRβ-Cre mice. (a) Immunofluorescence of skeletal muscle sections from uninjured mTmG;PDGFRβ-Cre mice with antibodies to CD34 and PDGFRα (Scale bar 30μm). (b) Immunofluorescence of skeletal muscle sections from CTX injured mTmG;PDGFRβ-Cre mice with antibodies to CD34 and PDGFRα (Scale bar 30μm). Flow cytometric analysis of (c) PDGFRα and (d) CD34 expression by GFP^+ reporter cells from uninjured skeletal muscle of mTmG;PDGFRβ-Cre mice (n = 3). Flow cytometric analysis of (e) PDGFRα and (f) CD34 expression by GFP^+ reporter cells from injured skeletal muscle of mTmG;PDGFRβ-Cre mice (n = 3).

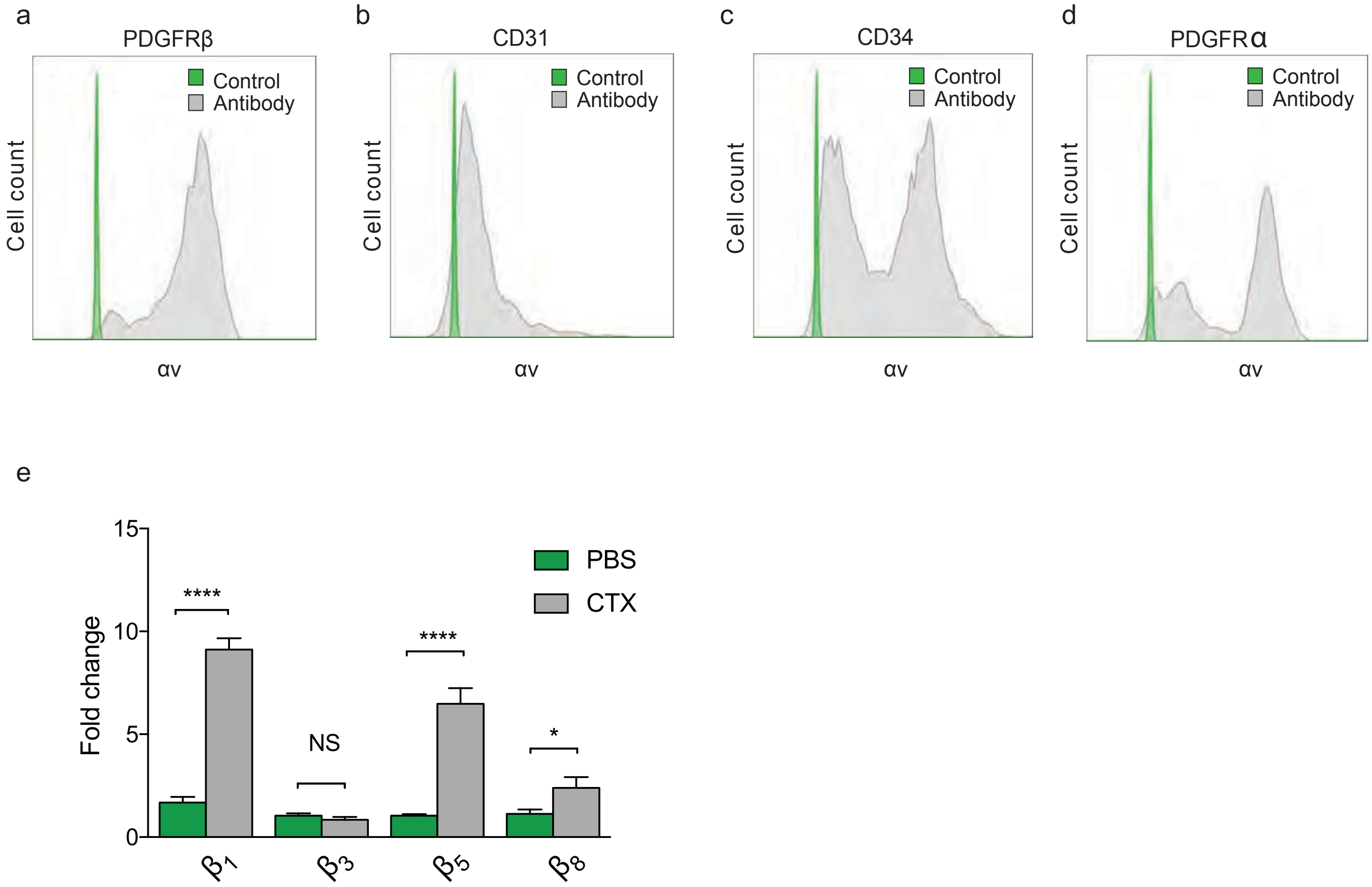

Supplementary Figure 3. αv integrin expression on (a) PDGFRβ⁺, (b) CD31⁺, (c) CD34⁺ and (d) PDGFRα⁺ populations in uninjured skeletal muscle. (e) Expression of αv integrin β-subunits in freshly sorted PDGFRβ⁺ reporter cells from control (PBS) and fibrotic (CTX) skeletal muscle (n=8). Data are mean ± s.e.m. *$p<0.05$, ****$p<0.0001$ (Student's t-test).

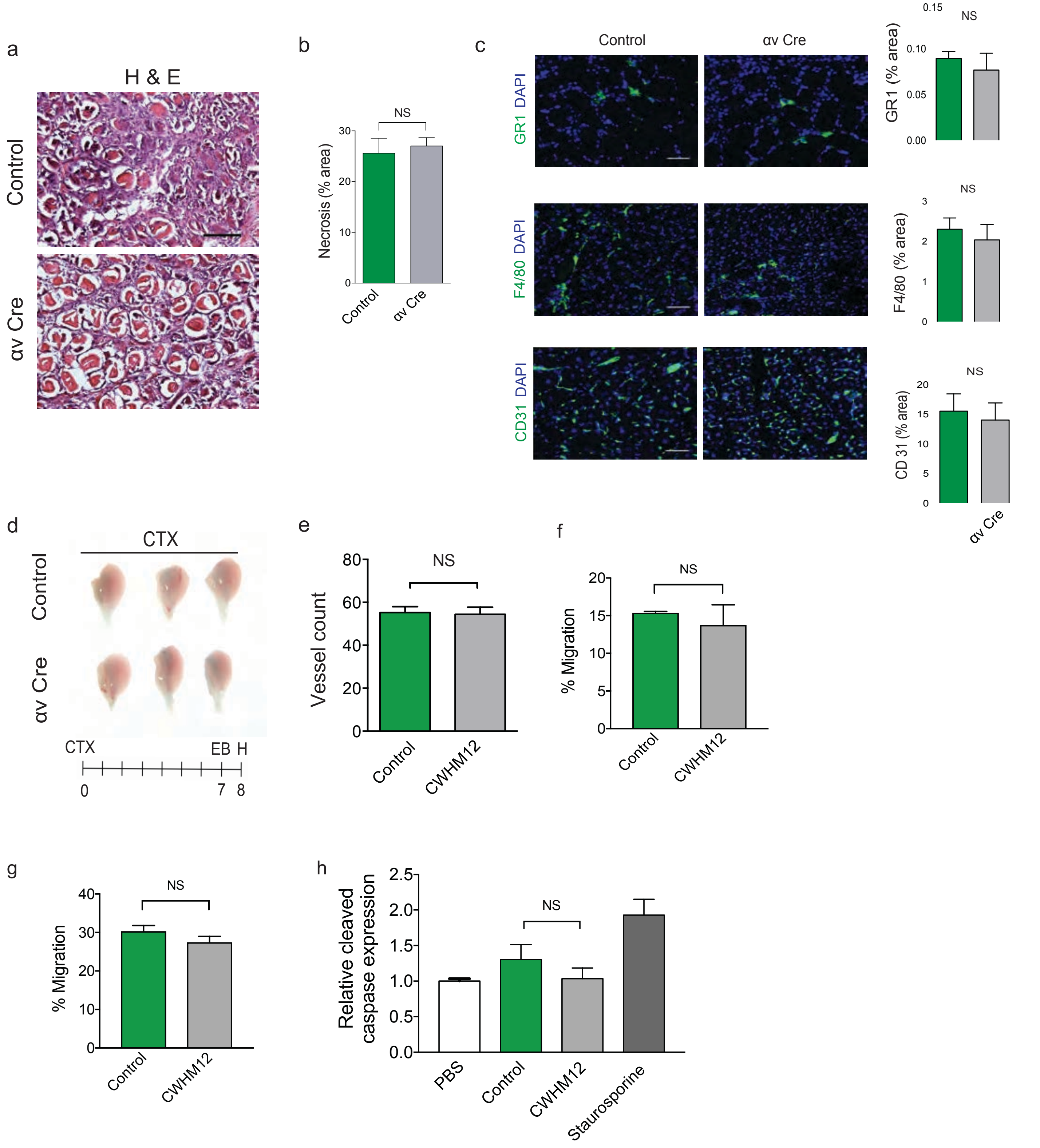

Supplementary Figure 4. αv integrin depletion does not influence **tissue necrosis, the inflammatory response or neovascularlisation following CTX-induced muscle injury or apoptosis and migration of PDGFRβ+ cells in vitro.** (a) Representative images of haematoxylin and eosin staining in control and αv Cre mice at day 8 post CTX injection. Scale bar 100μm. (b) Digital image analysis quantification of haematoxylin and eosin staining in control and αv Cre mice 8 days post CTX or control (PBS) injection (n=5). (c) Gr1, F4/80 and CD31 immunohistochemistry of skeletal muscle tissue harvested 8 days after CTX treatment of control and αv Cre mice. Scale bars 50μm. Digital image analysis quantification of staining (n=4). (d) Representative tibialis anterior muscles harvested 8 days after CTX-induced injury and 1 day after intraperitoneal Evans Blue injection. (e) Quantification of vascularity (vessel count) of skeletal muscle harvested 21 days following CTX treatment of control and Itgavflox/flox-;PDGFRβ-Cre mice. (f) Migration of PDGFRβ^{+} cells from skeletal muscle at 4 hours and at (g)18 hours post treatment with control enantiomer or CWHM12. (f) Apoptosis of PDGFRβ^{+} cells from skeletal muscle treated with PBS, control enantiomer, CHWM12 or staurosporine as determined by cleaved caspase activity. Data are mean ± s.e.m. (Student´s t-test).

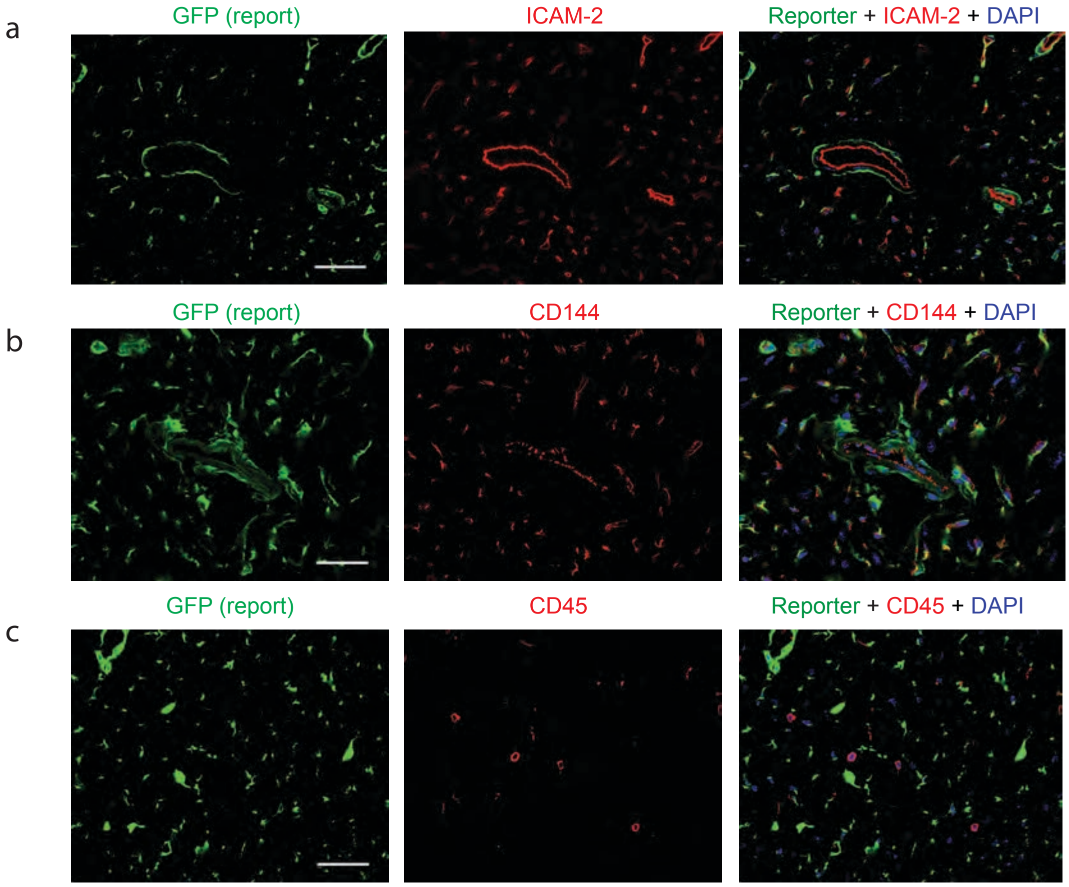

Supplementary Figure 5. Immunofluorescence micrographs of cardiac muscle sections from mTmG;PDGFR β-Cre reporter mice (n=4) stained with (a) anti-CD144, (b) anti-ICAM-2 and (c) anti-CD45 (scale bar 30μm).

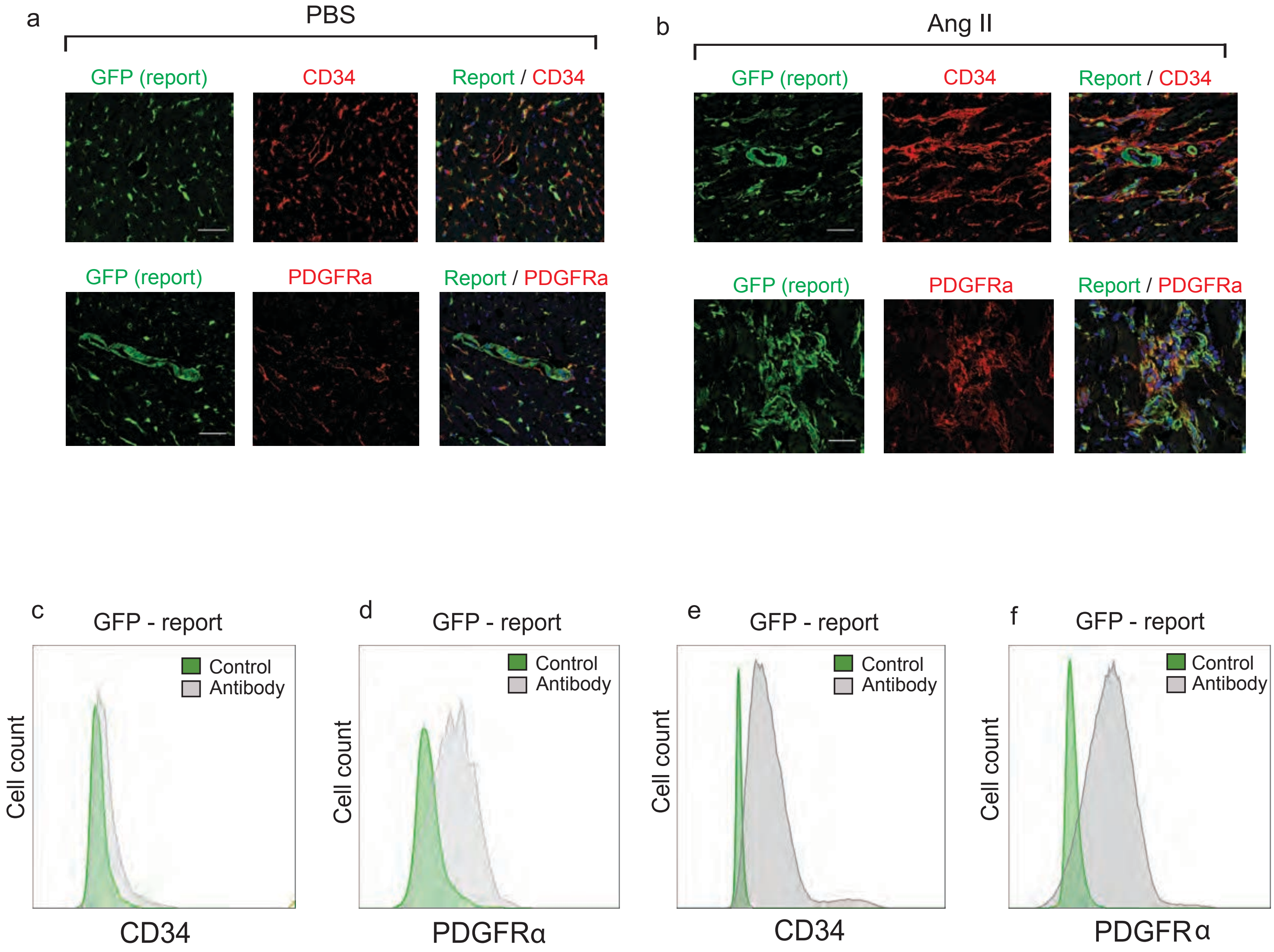

Supplementary Figure 6. Expression of PDGFRα and CD34 in PDGFRβ$^+$ reporter cell populations from mTmG;PDGFRβ-Cre mice. (a) Immunofluorescence of cardiac muscle sections from uninjured mTmG;PDGFRβ-Cre mice with antibodies to CD34 and PDGFRα. (b) Immunofluorescence of cardiac muscle sections from AngII injured mTmG;PDGFRβ-Cre mice with antibodies to CD34 and PDGFRα. Flow cytometric analysis of (c) CD34 and (d) PDGFRα expression of GFP$^+$ reporter cells from cardiac muscle from uninjured mTmG;PDGFRβ-Cre mice. Flow cytometric analysis of (e) CD34 and (f) PDGFRα expression of GFP$^+$ reporter cells from cardiac muscle from AngII injured mTmG;PDGFRβ-Cre mice.

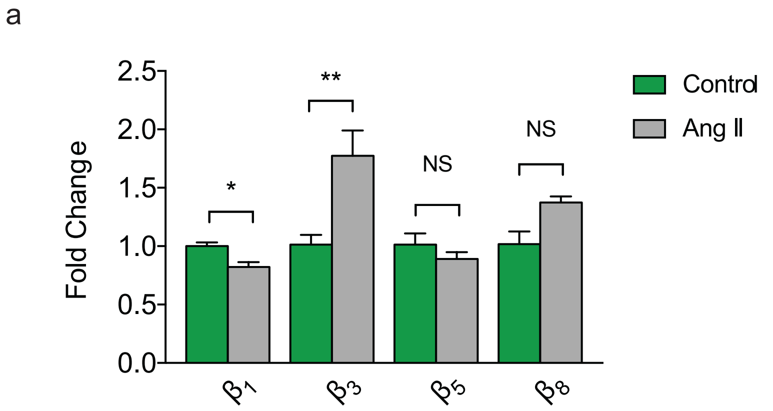

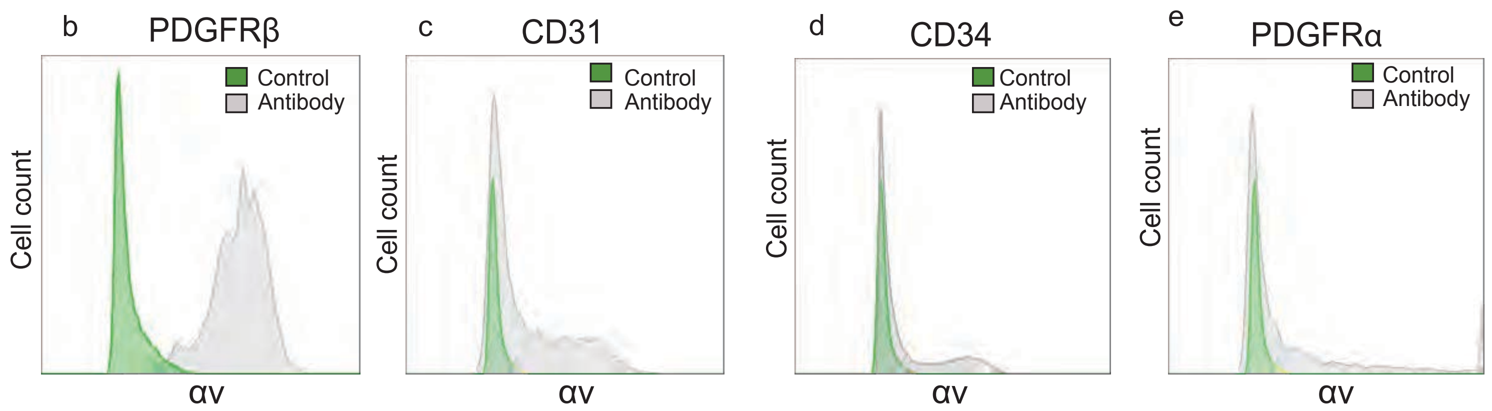

Supplementary Figure 7. (a) Expression of αv integrin β-subunits in freshly sorted PDGFRβ⁺ reporter cells from control and fibrotic (Ang II) cardiac muscle (n=8). Flow cytometric analysis of αv integrin expression on (b) PDGFRβ⁺, (c) CD31⁺, (d) CD34⁺ and (e) PDGFRα⁺ populations in uninjured cardiac muscle (n=3). Data are mean ± s.e.m. *p<0.05, **p<0.01 (Student´s t-test).

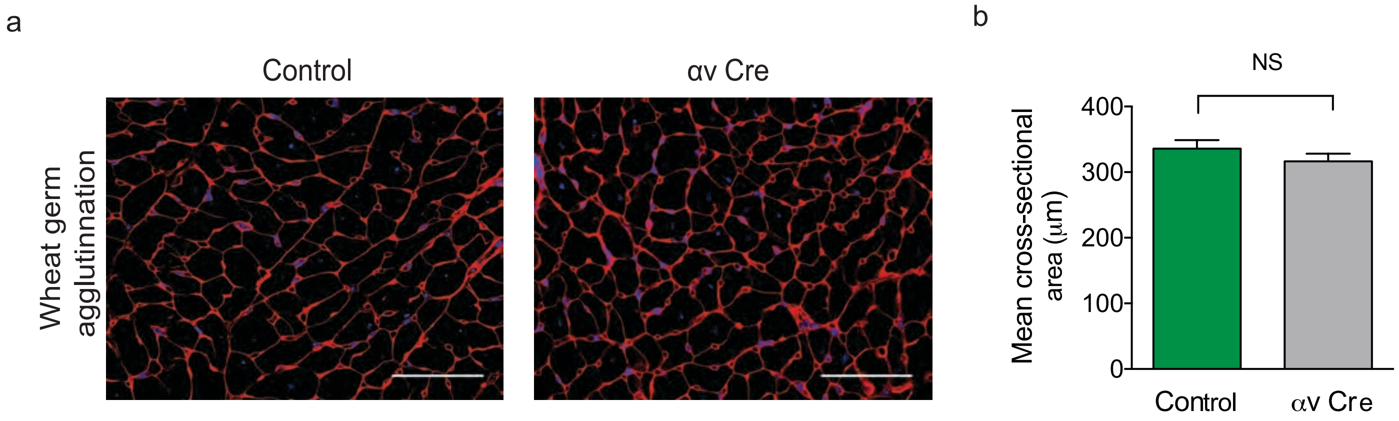

Supplementary Figure 8. Cardiomyocyte cross sectional area assessment in hearts from control and Itgavflox/flox;PDGFRβ-Cre mice. (a) Representative images of wheat germ agglutinin and dapi stained sections of cardiac muscle from control and Itgavflox/flox;PDGFR β-Cre mice treated for 14 days with 200ng/kg/min Angiotensin II. Scale bar 50μm (b) Digital image analysis quantitation of cardiac hypertrophy determined by cross-sectional area of cardiomyocytes with central nuclei.

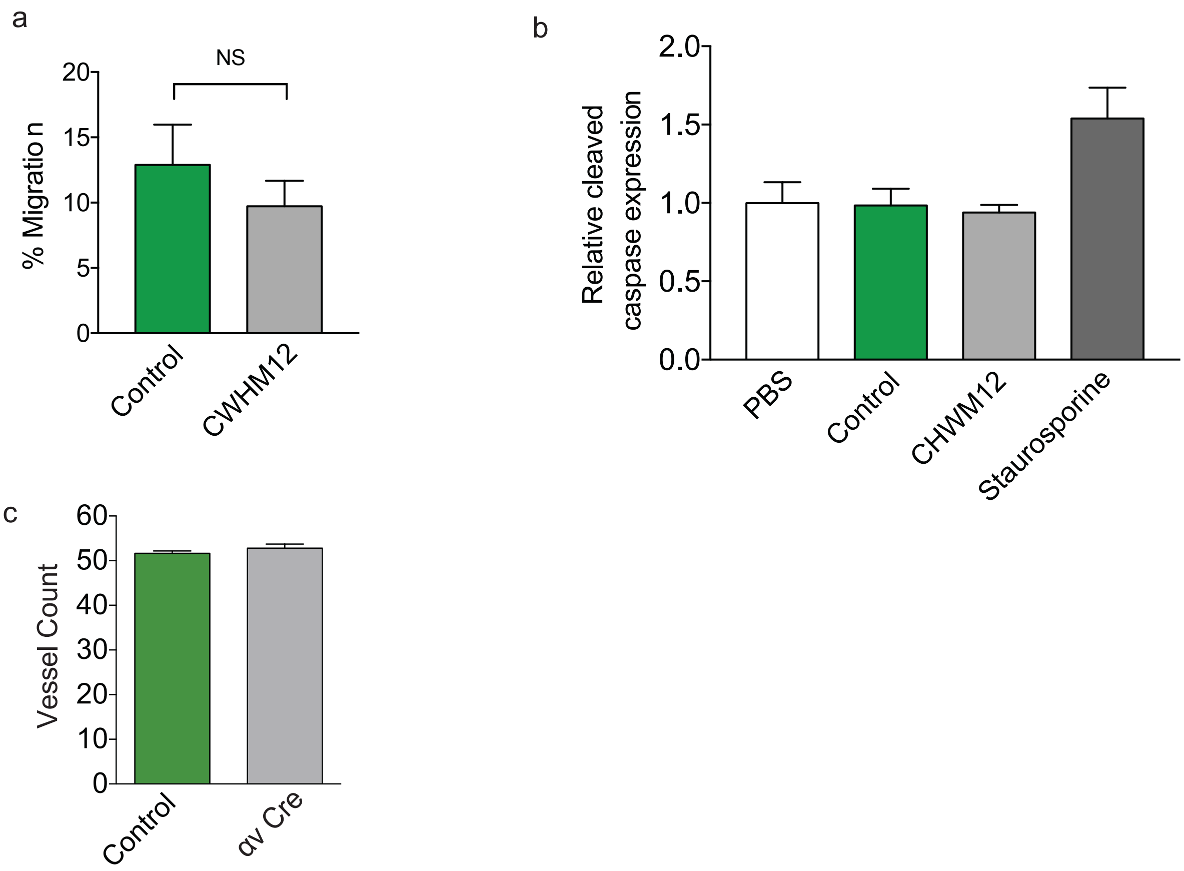

Supplementary Figure 9. Pharmacologic blockade of αv integrins does not influence apoptosis or migration of PDGFRβ⁺ cells *in vitro* or neovascularisation of cardiac muscle following 14 days of Angiotensin II treatment. (a) Migration of cardiac muscle PDGFRβ⁺ cells treated with control enantiomer and CHWM12. (b) Apoptosis of PDGFRβ⁺ cells from cardiac muscle treated with PBS, control enantiomer, CHWM12 or staurosporine determined by cleaved caspase activity. (c) Quantitation of vascularity (vessel counting) of cardiac muscle harvested at 14 days Angiotensin II treatment of control and Itgavflox/flox;PDGFRβ-Cre mice.Data are mean ± s.e.m. (Student´s t-test).

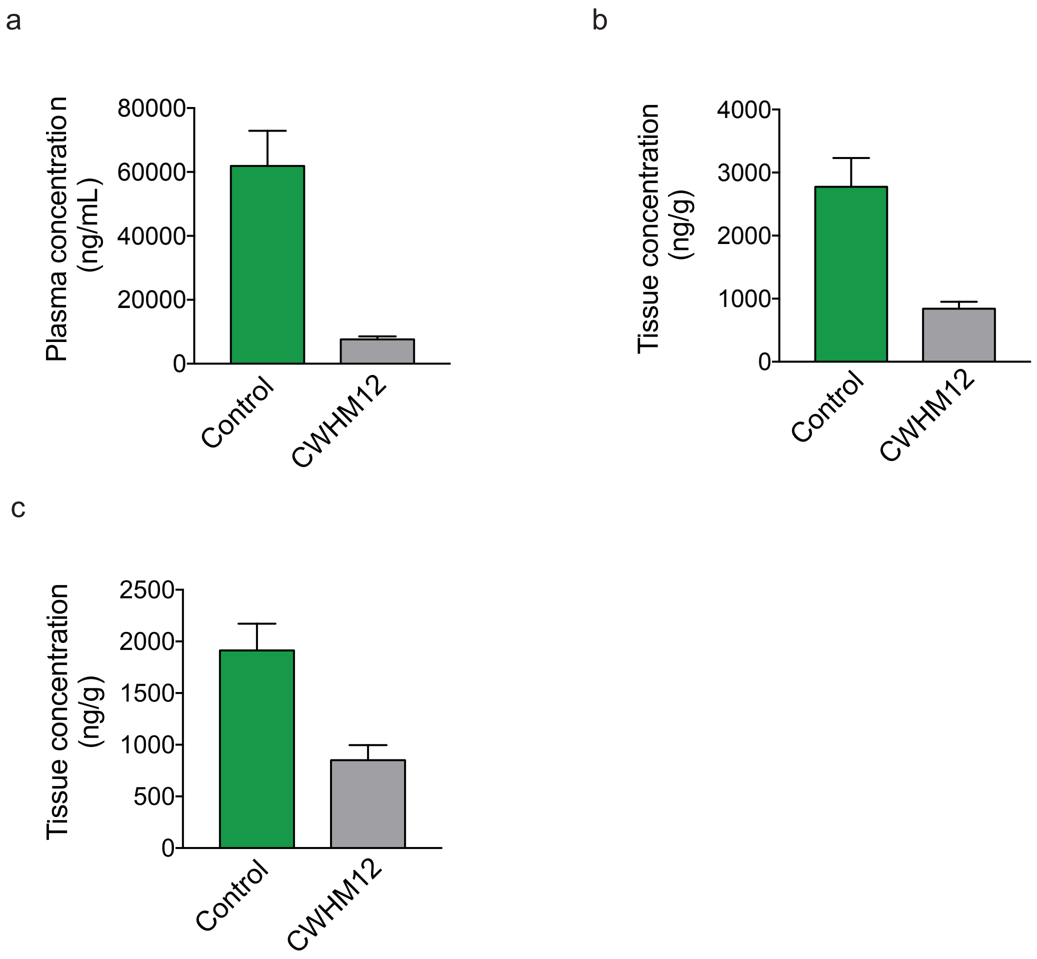

Supplementary Figure 10. Levels of CWHM12 and control (CWHM96) enatiomer in plasma, skeletal muscle and cardiac muscle following subcutaneous osmotic mini-pump delivery. (a) Plasma, (b) skeletal muscle and (c) cardiac muscle concentrations of CHWM12 and control enantiomer following 3 days of continuous minipump delivery at 100 mg/kg/day (n=5). Data are expressed as mean ± s.e.m.

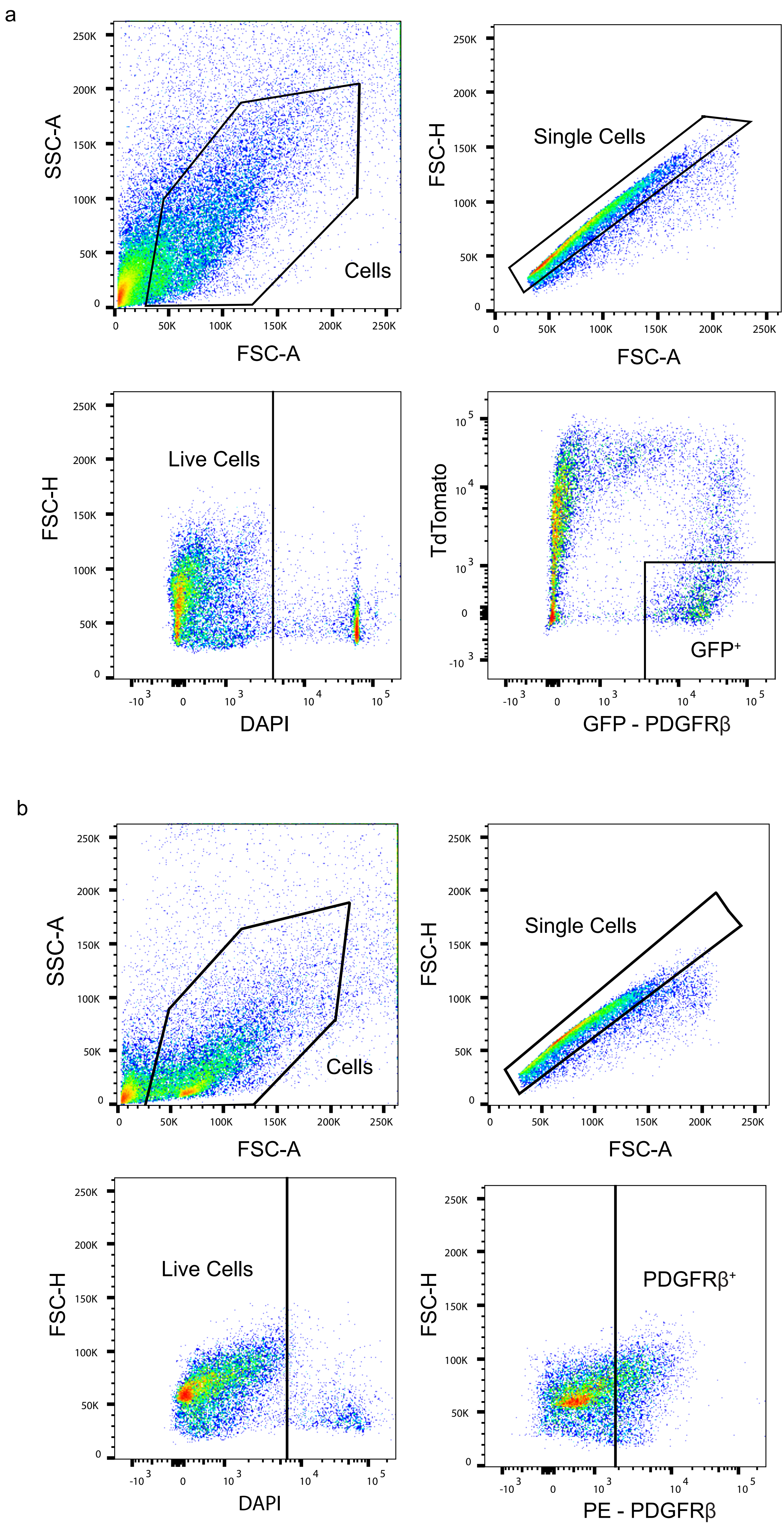

Supplementary Figure 11. Fluorescent activated cell sorting (FACS) gating strategy used to isolate PDGFRβ$^+$ populations from mTmG;PDGFRβ-Cre and non-reporter tissue. (a) FACS isolation of GFP$^+$ cells from mTmG;PDGFRβ-Cre skeletal muscle. A side scatter area (SSC-A) versus forward scatter area (FSC-A) plot was used to remove debris, then a forward scatter height (FSC-H) versus FSC-A plot was used to isolate single cells. DAPI positive cells were eliminated prior to collecting GFP$^+$ TdTomato$^-$ cells (b) FACS isolation of PDGFRβ$^+$ cells from non-reporter mouse skeletal muscle. A side scatter area (SSC-A) versus forward scatter area(FSC-A) plot was used to remove debris, then a forward scatter height (FSC-H) versus FSC-A plot was used to isolate single cells. DAPI positive cells were eliminated prior to collecting PDGFRβ$^+$ cells.
